# De novo transcriptome assembly and annotation of the third stage larvae of the zoonotic parasite *Anisakis pegreffii*

**DOI:** 10.1186/s13104-022-06099-9

**Published:** 2022-06-25

**Authors:** Marialetizia Palomba, Pietro Libro, Jessica Di Martino, Aurelia Rughetti, Mario Santoro, Simonetta Mattiucci, Tiziana Castrignanò

**Affiliations:** 1 Department of Ecological and Biological Sciences, Tuscia University, Viale dell’Università s/n, 01100 Viterbo, Italy; 2 Department of Experimental Medicine, “Sapienza” University of Rome, P.le Aldo Moro, 5, 00185 Rome, Italy; 3 Department of Integrative Marine Ecology, Stazione Zoologica Anton Dohrn, Villa Comunale, 1, 80121 Naples, Italy; 4 Department of Public Health And Infectious Diseases, Section of Parasitology, Sapienza University of Rome, P.le Aldo Moro, 5, 00185 Rome, Italy; 5 Laboratory affiliated to Istituto Pasteur Italia—Fondazione Cenci Bolognetti, Rome, Italy

**Keywords:** *Anisakis pegreffii*, Zoonotic parasite, Transcriptome, De novo assembly, Gene annotation

## Abstract

**Objectives:**

*Anisakis pegreffii* is a zoonotic parasite requiring marine organisms to complete its life-history. Human infection (anisakiasis) occurs when the third stage larvae (L3) are accidentally ingested with raw or undercooked infected fish or squids. A new de novo transcriptome of *A. pegreffii* was here generated aiming to provide a robust bulk of data to be used for a comprehensive "ready-to-use" resource for detecting functional studies on genes and gene products of *A. pegreffii* involved in the molecular mechanisms of parasite-host interaction.

**Data description:**

A RNA-seq library of *A. pegreffii* L3 was here newly generated by using Illumina TruSeq platform. It was combined with other five RNA-seq datasets previously gathered from L3 of the same species stored in SRA of NCBI. The final dataset was analyzed by launching three assembler programs and two validation tools. The use of a robust pipeline produced a high-confidence protein-coding transcriptome of *A. pegreffii*. These data represent a more robust and complete transcriptome of this species with respect to the actually existing resources. This is of importance for understanding the involved adaptive and immunomodulatory genes implicated in the “cross talk” between the parasite and its hosts, including the accidental one (humans).

## Objective

*Anisakis pegreffii* is a parasitic nematode belonging to the *A. simplex* (s.l.) species complex [1, 2]. It has a heteroxenous life cycle involving mainly cetaceans as definitive hosts, crustaceans as first intermediate hosts, fish, and squids as intermediate/paratenic ones. Its geographical distribution includes the Mediterranean Sea, the Iberian Atlantic coast waters, and the Austral region waters, between 30°S and 60°S. In humans, the accidental ingestion of third-stage larvae (L3) through the consumption of infected raw, undercooked, or improperly processed fish, causes a zoonosis, known as anisakiasis. Among the currently recognized nine biological species of the genus, so far only *A. pegreffii* and *A. simplex* (s.s.) cause anisakiasis [1, 3, 4].

The investigation of genes and proteins of *A. pegreffii* is crucial for understanding the parasite biological functions and its adaptation to abiotic and biotic conditions. It also represents a fundamental aspect to add knowledge about the molecular mechanisms involved in the evolutionary host-parasite interaction. Additionally, the molecules involved in the interaction between *A. pegreffii* and humans have not yet been elucidated. Finally, the absence of a suitable reference genome of this parasite species could make it difficult to achieve those goals. Although several RNA-seq analyses of L3 *A. pegreffii* at different experimental conditions and from different larvae tissues were carried out [5–9], a complete “ready to use” transcriptome is missing.

Objective of this research was to provide a robust high-confidence protein-coding transcriptome of the L3 stage of *A. pegreffii* acquired from the assembly of data newly generated in the present study with those previously stored. The findings were to provide a more accurate de novo reference transcriptome of *A. pegreffii* that will allow to shed light on genes implicated in the "cross-talk" between the parasite and its natural and accidental hosts.

## Data description

The input dataset for de novo assembly of *A. pegreffii* L3 was composed by six RNA-seq datasets (Table 1, Data file 1, 2): one obtained in the present study (PRJNA752284) (Table 1, Data file 2) and five retrieved from SRA of NCBI (PRJNA589243, PRJNA602791, PRJNA374530, PRJNA316941, PRJNA312925). In order to obtain the RNA-seq dataset in this study, A*. pegreffii* L3, collected from the viscera of fish from the Mediterranean Sea, were maintained in vitro culture for 24 h. RNA and DNA were extracted from nine L3 using TRIzol reagent, as previously described [10, 11]. The extracted RNA from each three L3 was pooled, and the quantity check was performed by using Agilent 2100 Bioanalyzer. The cDNA library was prepared using the TruSeq Stranded mRNA kit (Illumina). Ligated products of 200 bp were excised from agarose gels and PCR amplified. Products were single end sequenced on an Illumina TruSeq platform. Genetic/molecular identification of L3 *A. pegreffii* was performed by sequences analysis of mitochondrial (mtDNA *cox*2), and nuclear (EF1 *α* − 1 nDNA, *nas* 10 nDNA) gene loci, as previously described [12﻿].

Bioinformatic analysis was performed using a High-Performance-Computing platform [13]. For each bioproject, the quality control of reads was performed running FastQC v.0.11.2, before and after trimming step (Trimmomatic v.0.39 [14]). The quality assessment metrics for all trimmed data were aggregated with MultiQC v.1.9 [15]. Data file 3 (Table 1) shows both the mean read counts per quality scores and the mean quality scores in each base position higher than 35, for all the samples in the six analyzed bioprojects. A total of 393,512,048 cleaned reads (97% of whole raw reads) were obtained after the removal of the low-quality reads.

In order to construct a robust de novo transcriptome, three assembly tools with a multi-kmer approach were adopted: Trinity v.2.11.0 [16] (Table 1, Data file 4), rnaSPAdes v.3.14.1 [17] (Table 1, Data file 5) and Oases v.0.2.09 [18] (Table 1, Data file 6). Results for each assembler were merged with Transabyss v.2.0.1 [19] (Table 1, Data file 7). The merged assembly of *A. pegreffii* showed an average length of 939 bp and an N50 of 2859 bp. The assembly was validated with two algorithms: Busco v.4.1.4 [20] and Transrate v.1.0.3 [21]. A CD-HIT-est run v.4.8.1 was applied to the merged assembly to remove any redundant transcripts. A total of 394,635 unique genes were provided (Table 1, Data file 8) and a quality check was re-applied. A total of 260,872 ORFs were predicted by using Transdecoder v.5.5.0 [22] (Table 1, Data file 9).

The functional annotation of contigs was performed by using DIAMOND v.2.0.11 [23], calling both blastp and blastx functions against three databases (Nr, SwissProt and TremBL). The obtained results for blastx consisted in 86,982 (88.93%), 56,997 (58.47%) and 87,134 (89.39%) sequences against Nr (Table 1, Data file 10), SwissProt (Table 1, Data file 11) and TremBl (Table 1, Data file 12), respectively. Mapped transcripts listed in the Data file 10, yielded 38,972 matches (hits) with *A. simplex*. Blastp results also are available for Nr (Table 1, Data file 13), SwissProt (Table 1, Data file 14) and TremBl (Table 1, Data file 15). Output from InterProScan used to annotate protein signatures is available in Data file 16 (Table 1). In detail, 18,976 contigs were annotated: 5099 GO-annotated and 2800 KEGG-annotated.

**Table 1:** Overview of data files/data sets

Label	Name of data file/data set	File types (file extension)	Data repository and identifier (DOI or accession number)
Data file 1	RNA-seq datasets from NCBI	Table file (.doc)	Figshare https://doi.org/10.6084/m9.figshare.19174214 [24]
Data file 2	RNA-seq dataset obtained in this study	Fastq file (.fastq)	NCBI https://identifiers.org/ncbi/bioproject:PRJNA752284 [25]
Data file 3	MultiQC reads quality results	Image file (.jpg)	Figshare https://doi.org/10.6084/m9.figshare.18480635 [26]
Data file 4	Trinity RNA-seq de novo transcriptome assembly	Fasta file (.fasta)	Figshare https://doi.org/10.6084/m9.figshare.18300896 [27]
Data file 5	rnaSPAdes RNA-seq de novo transcriptome assembly	Fasta file (.fasta)	Figshare https://doi.org/10.6084/m9.figshare.18301337 [28]
Data file 6	Oases RNA-seq de novo transcriptome assembly	Fasta file (.fasta)	Figshare https://doi.org/10.6084/m9.figshare.18480689 [29]
Data file 7	*Anisakis pegreffii* RNA-seq de novo transcriptome assembly	Fastq file (.fastq)	ENA https://identifiers.org/ena.embl:ERZ5400090 [30]
Data file 8	Unigenes	Fasta file (.fasta)	Figshare https://doi.org/10.6084/m9.figshare.18301772 [31]
Data file 9	Open reading frames (ORFs) prediction	Fasta file (.fasta)	Figshare https://doi.org/10.6084/m9.figshare.18302102 [32]
Data file 10	Functional annotation from non-redundant (nr) NCBI	Text file (.txt)	Figshare https://doi.org/10.6084/m9.figshare.18295190 [33]
Data file 11	Functional annotation from Swiss-Prot	Text file (.txt)	Figshare https://doi.org/10.6084/m9.figshare.18295970 [34]
Data file 12	Functional annotation from TrEMBL UniProt	Text file (.txt)	Figshare https://doi.org/10.6084/m9.figshare.18296603 [35]
Data file 13	Functional annotation from non-redundant (nr) protein NCBI	Text file (.txt)	Figshare https://doi.org/10.6084/m9.figshare.18296933 [36]
Data file 14	Functional annotation from Swiss-Prot Protein	Text file (.txt)	Figshare https://doi.org/10.6084/m9.figshare.18297410 [37]
Data file 15	Functional annotation from TrEMBL UniProt Protein	Text file (.txt)	Figshare https://doi.org/10.6084/m9.figshare.18297938 [38]
Data file 16	InterProScan results	Text file (.txt)	Figshare https://doi.org/10.6084/m9.figshare.18298319 [39]

## Limitations

The *A. pegreffii* transcriptome here obtained was assembled with those RNA-seq data sets from the third larval stage of the parasite species. The single transcriptome available from the fourth stage larva of *A. pegreffii* [8] was not included in this analysis because the main aim of this analysis was to provide a robust and "ready to use'' transcriptome of the infective stage (third larval stage) of the parasite also provoking the zoonotic disease (anisakiasis) to humans.

## Data Availability

The data described in this Data note can be freely and openly accessed on NCBI, ENA and figshare databases. Data from the following sex Bioprojects were used: PRJNA752284, PRJNA589243, PRJNA602791, PRJNA374530, PRJNA316941, PRJNA312925. The RNA-seq raw data here obtained have been deposited at ENA (ERZ54000). The other data files generated in the current study are available in the figshare database. Please see Table 1 and references [24–39] for details and links to the data.

## Abbreviations

L3: Third stage larvae
SRA: Sequence Read Archive
NCBI: National Center for Biotechnology Information
BUSCO: Benchmarking Universal Single-Copy Orthologs
Gb: Giga base-pair
bp: Base pair
